# Weld Feature Extraction Based on Semantic Segmentation Network

**DOI:** 10.3390/s22114130

**Published:** 2022-05-29

**Authors:** Bin Wang, Fengshun Li, Rongjian Lu, Xiaoyu Ni, Wenhan Zhu

**Affiliations:** College of Mechanical and Electronic Engineering, Nanjing Forestry University, Nanjing 210037, China; wbin@njfu.edu.cn (B.W.); 18980004560@njfu.edu.cn (F.L.); xiaoyuni@njfu.edu.cn (X.N.); zhuwenhan@njfu.edu.cn (W.Z.)

**Keywords:** deep learning, semantic segmentation, laser welding, seam tracking

## Abstract

Laser welding is an indispensable link in most types of industrial production. The realization of welding automation by industrial robots can greatly improve production efficiency. In the research and development of the welding seam tracking system, information on the position of the weld joint needs to be obtained accurately. For laser welding images with strong and complex interference, a weld tracking module was designed to capture real-time images of the weld, and a total of 737, 1920 × 1200 pixel weld images were captured using the device, of which 637 were used to create the dataset, and the other 100 were used as images to test the segmentation success rate. Based on the pixel-level segmentation capability of the semantic segmentation network, this study used an encoder–decoder architecture to design a lightweight network structure and introduced a channel attention mechanism. Compared to ERF-Net, SegNet, and DFA-Net, the network model in this paper has a fast segmentation speed and higher segmentation accuracy, with a success rate of 96% and remarkable segmentation results.

## 1. Introduction

Laser welding is a common process in industrial production today, and full automation of welding will greatly improve production efficiency. In order to improve the quality of welding and to eliminate deviation of the actual welding trajectory from the preset trajectory due to factors such as workpiece clamping errors and thermal deformation of the workpiece during laser welding, a seam tracking system needs to be designed to obtain real-time information on the position of the weld during welding operations. Laser vision sensing technology [1] is a hot topic in the field of automatic welding, in which a laser is projected onto the surface of the weld to form different forms of weld seam feature stripes, and then the features in an image are analyzed and processed. However, the welding site is complex, and the images collected during the operation are subject to different forms and degrees of interference, such as arc light, spatter, exposure, and fume, so the problem of the difficult extraction of weld laser line feature stripes becomes one of the difficult problems to be overcome by the weld tracking system.

Currently, segmentation processing of welding images is mainly divided into traditional image processing algorithms and machine learning methods. Many traditional image processing methods can extract weld feature stripes by filtering the image and then designing an image thresholding segmentation algorithm for a specific scene. Ye et al. [2] proposed an open operation and OSTU algorithm approach to the noise reduction processing of weld images to obtain accurate data of the weld seam. Shao et al. [3] used an acousto-optic modulator (AOM) to modulate the intensity of structured light and then used Fourier transform to filter out noise interference in the frequency domain. Zou et al. [4] used morphological methods and Gaussian kernel correlation algorithms to obtain the position of the feature points before and after welding, respectively. Although these traditional algorithms are fast, they are highly specific to the application and are not suitable for heavily disturbed welding images.

In recent years, deep learning techniques have become more mature, and semantic segmentation allows for the pixel-level classification of images. The development and widespread use of convolutional neural networks have enabled them to perform well in segmentation tasks, and their segmentation accuracy for complex tasks has surpassed that of traditional processing methods. Zhao et al. [5] used ERF-Net and a regression algorithm to extract weld feature points from a strongly disturbed background with an offset error of less than 0.25 mm from the manually annotated weld position. Zou et al. [6] proposed a method based on conditional generative adversarial networks to achieve the restoration of strongly disturbed weld images, and the final error of the method was stable within 0.2 mm. In the laser welding streak segmentation task in this paper, the acquired images are also heavily disturbed, so for the choice of method for the segmentation task, it is more appropriate to use a convolutional neural network. However, because of the real-time requirements of the welding task, there is a trade-off between segmentation speed and segmentation accuracy in the selection of a semantic segmentation network. In the actual welding work site, the computing power and memory size of the working machine are limited, and it is impossible to have the graphics processing unit (GPU) resources of a laboratory-level workstation. Excessive reduction in the parameters of the model or a reduction in network computation will result in a decrease in the segmentation accuracy, resulting in failure to extract weld feature points.

The main contributions of the work in this paper are as follows: (a) a tracking module in the weld tracking system was designed to capture images of the weld site, and (b) a semantic segmentation network is proposed for complex and disturbed weld images. The segmentation network designed in this study can segment laser welding seam feature stripes more quickly and accurately in complex and disturbed welding images, creating conditions for the subsequent development of a weld seam tracking system based on deep learning. This is of great significance and reference value for the practical application of proven deep learning techniques to the laser welding field.

## 2. Materials and Methods

### 2.1. Description of Weld Feature Extraction

**Figure 1:**
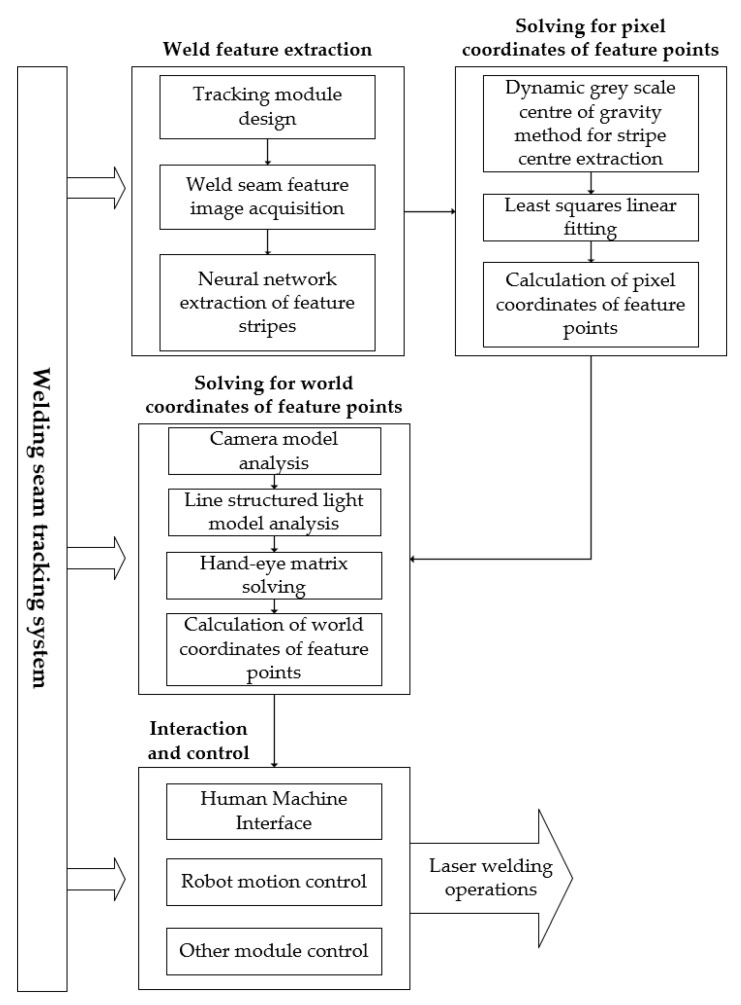
Overview of the weld streak extraction section in the weld tracking system.

Weld feature extraction is an important part of the weld tracking system (Figure 1), and the quality of the feature stripe extraction affects the subsequent calculation of the pixel coordinates of the weld point on the picture. The world coordinates of the feature points can be found by performing a model analysis of the pinhole camera and the line structured light plane and by solving the hand–eye matrix (the coordinate conversion matrix between the robot arm and the camera fixed to the arm). The robot then adjusts the laser gun position for the welding operation according to the error between the actual world coordinates and the preset trajectory, and it can even perform adaptive laser welding tasks without a preset welding trajectory.

This paper investigates the extraction of feature stripes from a V-shaped weld, where the bottom tip of the “V” is the weld point to be welded. Since there is a large and strong background light interference during welding, a neural network can be used to extract the feature stripes (generated by the line laser transmitter) from the images of the welding site in real time and then use some algorithms to calculate the coordinates of the actual welding point. The weld seam tracking system can then adjust the control of the industrial robot accordingly based on the information obtained about the actual weld joint coordinates.

### 2.2. Design of Weld Seam Tracking Module

**Figure 2:**
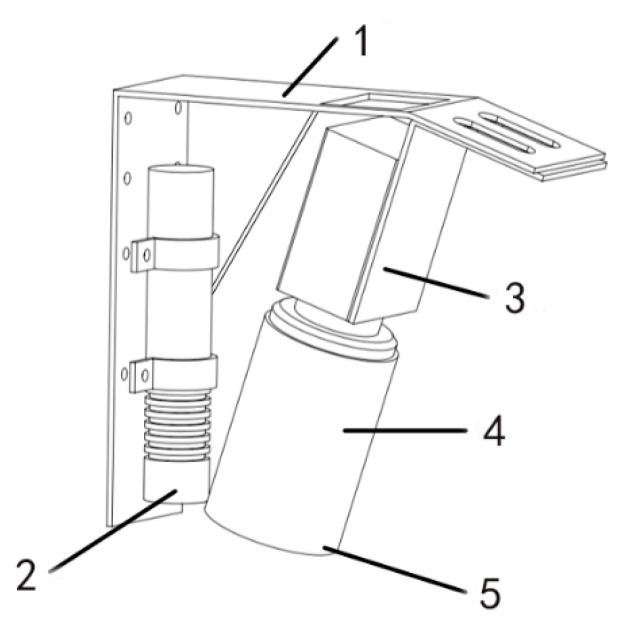
Weld tracking module. (**1**) Shell, (**2**) line laser transmitter, (**3**) industrial camera, (**4**) camera lens, and (**5**) optical filter.

**Figure 3:**
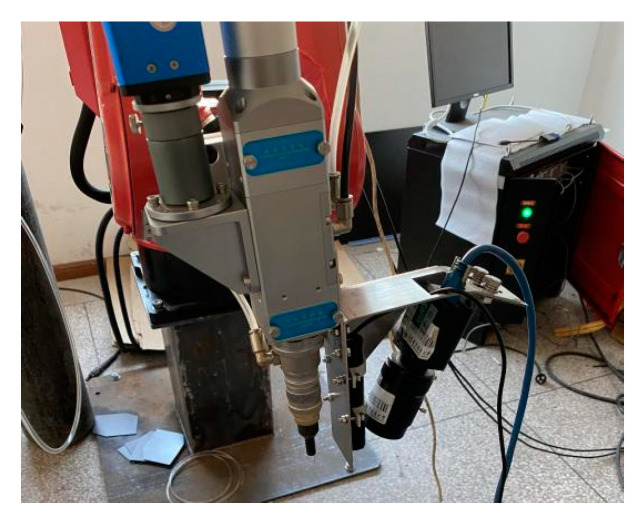
Tracking module installation location diagram.

In the laser welding seam recognition and tracking system, the seam tracking module is the core device. The seam tracking device designed in this work is shown in Figure 2, which mainly includes three parts: a fixed shell, a linear laser emitter, and an industrial camera. The fixing effect of the shell includes not only fixing the wire laser and camera but also fixing the tracking device near the laser welding joint at the end of the industrial robot arm. The specific installation position of the device is shown in Figure 3. The tracking module and the welding gun are fixed to the robot arm, and the stripes generated by a line laser emitter are positioned at a small fixed distance ahead of the welding gun at all times. In addition, the shell can also play a role in protecting the camera, and the metal plate can prevent the splash generated during welding. The laser emitter emits a linear plane laser to the surface of the weldment, and different welding stripe shapes can be generated on different types of weldments. The image containing laser stripes can be obtained by the camera on the surface of the weldment. Subsequently, the laser stripes of the weld feature in the image can be segmented by the neural network, which provides conditions for determining the pixel coordinates of the weld feature points from the stripes. The filter can filter part of the interference light in the welding process so that the camera can collect higher-quality images.

The linear laser transmitter model used in this study was an HO-S635PX-22110, and the red light wavelength emitted was 635 nm. The industrial camera was a Sony IMX174 with three color RGB channels, and the model was a Point Gray GS3-US-23S6C. The resolution of the collected image was 1920 × 1200, which needed to be transmitted by a USB 3.0 interface. The number of frames per second was 30, and the specific parameters are shown in Table 1.

### 2.3. Building the Dataset

**Figure 4:**
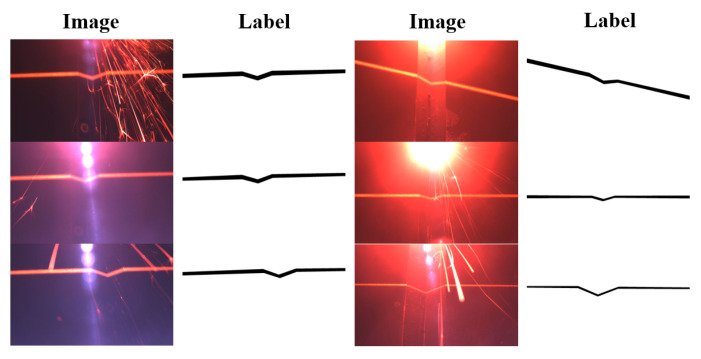
Part of the original and labeled images.

We collected a total of 737 welding scene images, of which 637 images were used to produce datasets, and the remaining 100 images (acquired again later) were reserved for segmentation for actual effect comparison. The labeling was completed by the LabelMe tool (developed by MIT, an online Javascript image annotation tool), after which the training, validation, and test sets were randomly divided in a ratio of 8:1:1 (Table 2), and their numbers were rounded to 509, 64, and 64, respectively. Some of the original and labeled images are shown in Figure 4. There is a distance between the welding gun and the camera, and the intense light produced during the laser welding process causes the brightest areas of interference in the images. The intensity of the light produced by the laser melting the weld varies, resulting in images with different backgrounds such as blue, red, and black. The red stripe with the “V” is formed by the light emitted by the line laser transmitter on the surface of the weld to help locate the weld joint (at the tip of the “V”) to be welded in the seam.

We did not perform any image processing (rotation, scaling, translation, etc.) on the images. Some of the feature stripes in the images are skewed because we rotated the line laser emitter at a certain angle during acquisition to take into account the diversity of the dataset samples. The size of the labeled images is 1920 × 1200 but was similarly reduced to 640 × 640 during training.

## 3. Design of a Segmentation Network

ERF-Net [7] is a semantic segmentation network with excellent performance and was proposed by Eduardo Romera. After extensive research, an encoder–decoder architecture was adopted and stacked using 1D convolutional layers with residual connections to solve the model degradation problem and reduce the computational cost. At the beginning of the design, the network architecture makes a good trade-off between segmentation efficiency and segmentation quality. Finally, the segmentation speed and segmentation accuracy are better than most similar networks. Due to its excellent performance, the Pytorch version of this network has been commercially adopted and is no longer open source. However, in the application of weld seam feature extraction, the network still needs to be improved. Therefore, the semantic segmentation network designed in this work learns the core convolution module of ERF-Net, that is, the 1D convolution block and residual connection structure. On this basis, lightweight improvements were made, and measures such as an attention mechanism were introduced to further improve the segmentation accuracy.

The segmentation network in this paper still follows the classic encoder–decoder architecture. The role of the encoder network is to extract important semantic information from the input image and generate feature tensors; the role of the decoder network is to restore the low-resolution feature tensor output from the encoder stage back to the original input size image for pixel-by-pixel classification. Compared to other multi-branch architectures, this encoder–decoder architecture is simple, straightforward, and fast. Ma et al. [8] (the authors of Shuffle-Net v2) proposed four principles for improving a lightweight network design, one of which is to limit the use of multi-branch structures. Due to the simple structure of the encoder–decoder architecture and the lack of other branch structures [9], the network inference speed of this architecture is relatively fast. The designed network in this paper also tries to adhere to these four improvement principles [8] but does sometimes unavoidably violate some of them.

**Figure 5:**
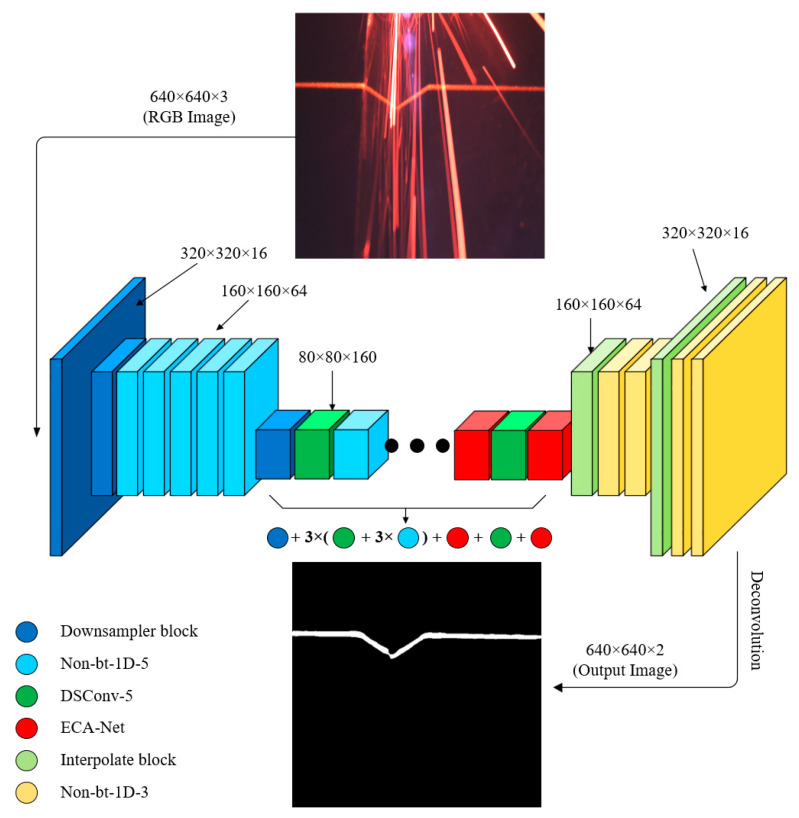
Overview of network architecture for end-to-end training.

On the basis of ERF-Net, we carried out lightweight improvements [10] and designed a more efficient (speed and accuracy) semantic segmentation neural network that is trained end-to-end and contains residual connection structures (shown in Figure 5), and the specific network structure is shown in Table 3.

The image is captured by the camera as a 1920 × 1200 size RGB image, but a larger image affects the inference speed of the network, so the image is reduced in size to 640 × 640 pixels before being passed into the network to facilitate training (reducing the image will not affect the accuracy of the segmentation later). The technique for reducing the images is to use the resize function in the cv2 library, which uses a bilinear interpolation method. The network outputs the same 640 × 640 image, which is then eventually restored to its original size.

In our architecture, the downsampling modules in layers 1–23 are different convolutional blocks from the encoder, and layers 24–30 are the decoder part. In the next section, the various convolution blocks, downsampling and upsampling modules, and other structures in the table are described in detail.

## 4. Modules in the Network

**Figure 6:**
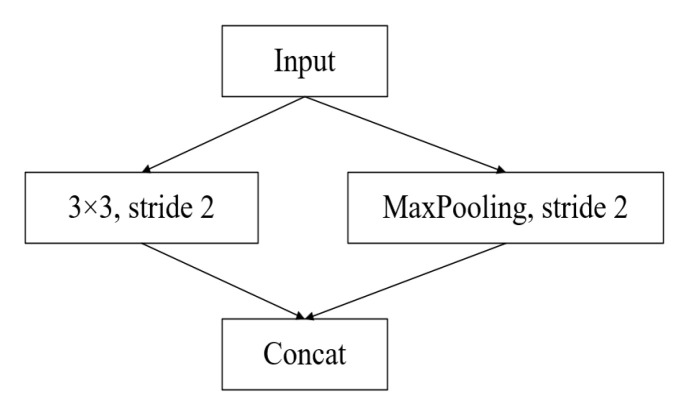
Downsampling block.

The downsampling operation, although reducing the pixel accuracy of the image, can help reduce the computational effort of the network while also helping to retain the main feature information for classification by the deeper network. The downsampling module of the network in this paper still uses the downsampling module in E-Net [11] (ERF-Net also adopts the downsampling module of E-Net), as shown in Figure 6. The input feature tensor is convolved by a convolution kernel of size 3 × 3 (step 2) and a maximum pooling operation, and the resulting feature maps are stacked together to obtain the output of a feature map with the width and height halved.

Since the advent of ResNet [12], almost all networks have included a residual structure. In our network structure, almost every layer contains a residual connectivity structure as well, and this residual connectivity largely reduces the problem of network degradation, as the convolutional layers keep stacking deeper. The residual connection structure enables the convolutional layer to perform residual learning, which is equivalently mapped through shortcuts:

(1) $$y=F\left(x,\{W_{i}\}\right)+x$$

(2) $$F=W_{2}\sigma \left(W,x\right)$$

where *x* denotes the layer input vector, *y* denotes the layer output vector, and $F\left(x,\left\{W_{i}\right\}\right)$ denotes the residual mapping to be learned. When *F*(*x*) is of different dimensions from *x*:

(3) $$y=F\left(x,\left\{W_{i}\right\}\right)+W_{s}x$$

where $W_{s}$ represents coefficients that linearly map *x* to match the dimensions.

**Figure 7:**
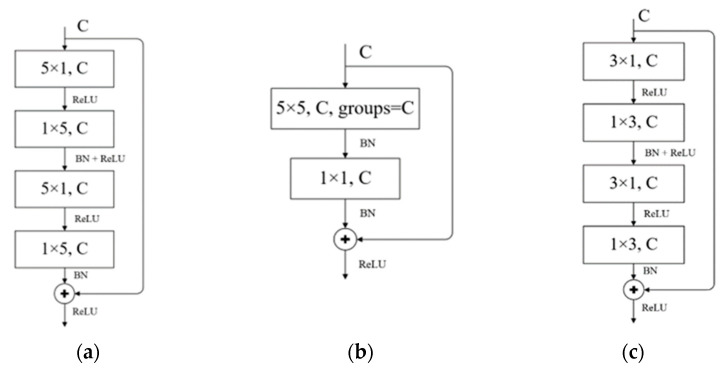
(**a**) Non-Bt-1D-5 module, (**b**) DSConv-5 module, and (**c**) Non-Bt-1D-3 module.

The Non-bt-1D-5 module is shown in Figure 7a (C in the figure represents the number of channels), which follows the core module of ERF-Net. The authors of ERF-Net believe that using 1D asymmetric convolution can significantly reduce the computational cost compared to conventional convolution and can achieve similar accuracy. The improved network in this paper therefore also uses this asymmetric 1D convolution instead of the conventional 2D convolution in the encoding stage, and it replaces the 3 × 1 and 1 × 3 convolution kernels with 5 × 1 and 1 × 5 kernels to obtain a larger perceptual field and to extract more information about the weld laser stripe features in the adjacent pixel space. In addition, if the convolutional layer uses the dilated convolution operation, dilated convolution is only used in the latter part of the two asymmetric 1D convolution operations. The group convolution in the network structure in this paper generally uses the maximum number of groups, and the group convolution operation is used for all 1D convolution blocks.

The use of a group convolution operation can effectively reduce the computation and the number of parameters, but the information interaction between the channels is missed. Therefore, in this paper, we used the random channel operation (channel split and channel shuffle) in ShuffleNet V2, a lightweight network, which can theoretically achieve weight sharing between the upper and lower layers and feature fusion between channels. However, in practice, we found that the network segmentation accuracy was not high and the inference speed was reduced after using the random channel strategy. In the end, we used depth-separable convolution with a convolutional kernel size of 5 × 5, which is often used in lightweight networks, and the structure of the DSConv-5 module is shown in Figure 7b.

Depth-separable convolution was proposed in Xception [13], divided into two parts: the first part is depthwise convolution, and the second part is pointwise convolution. Although the features between channels are still independent of one another in the channel-by-channel convolution, the second part of the point-by-point convolution performs a weighted combination in the depth direction through a 1 × 1 convolution kernel to complete the information interaction between channels. The network structure in this paper also focuses on fusing channel information through the second part instead of the role of the random channel strategy so that in the third stage of the encoder, the depth-separated convolutional modules are scattered and cross-distributed among the convolution layers.

**Figure 8:**
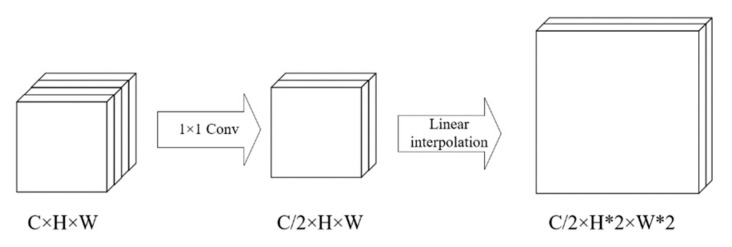
The upsampling module restores the input tensor process.

In the decoding stage, the decoder uses the Non-Bt-1D-3 module in ERF-Net (shown in Figure 7c). Unlike the larger convolution kernels in the Non-Bt-1D-5 module, we used smaller convolution kernels to obtain fine-grained image restoration and pixel classification. In the upsampling module, 1 × 1 convolution is used to reduce the number of channels of the input feature tensor, and then the image is restored (enlarged) by linear interpolation. The restoration process of the input tensor is shown in Figure 8.

To further improve the segmentation accuracy, we also introduced the relatively mature attention mechanism module. The attention mechanism [14] is the assignment of input weights and can be divided into channel, spatial, layer, mixed, and time domains according to the attention domain. The attention mechanisms in the spatial and channel domains are simpler and more frequently used, focusing respectively on the weight assignment to the number of channels and to the location information in space. As the welding task requires a certain degree of real-time performance and the spatial domain attention mechanism has a more complex network model with a higher number of parameters and computational cost, the simpler and less computationally intensive channel attention mechanism was finally chosen to improve the performance of the segmentation network by learning each channel and assigning different weights, suppressing the unimportant channels and focusing on the important ones.

Among the channel attention mechanisms, SE-Net [15] is widely used for its excellent performance as well as its plug-and-play feature. In the SE-Net network structure, a 1 × 1 convolutional kernel is used to reduce the number of parameters in order to perform dimensionality reduction, then a convolutional operation, and finally a 1 × 1 convolution to increase back to the original number of channels. However, the authors of ECA-Net [16] argued that this strategy destroys the direct correspondence between channels and attention weights and experimentally demonstrated a decrease in the effect of channel attention after dimensionality reduction.

The structure of ECA-Net was designed in such a way that the 1D convolution block sliding convolution processing of the resulting 1 × 1 × C feature map avoids the performance loss caused by the channel number transformation, yet it achieves the information interaction between the features in each channel. The length of the convolution kernel *k* of the 1D convolution has an adaptive type for different channel numbers, and the corresponding non-linear relationship between the number of channels *C* and the length of the convolution kernel *k* is shown in Equation (4), with the length *k* rewritten as Equation (5).

(4) $$C=\phi \left(k\right)=2^{\left(\gamma *k-b\right)}$$

(5) $$k=\psi \left(C\right)={\left|\frac{\log_{2}\left(C\right)}{\gamma }+\frac{b}{\gamma }\right|}_{odd}$$

where γ and *b* are the parameters of the linear relationship, γ is 2, *b* is 1, and ${\left|x\right|}_{odd}$ denotes the nearest odd number to *x*.

**Figure 9:**
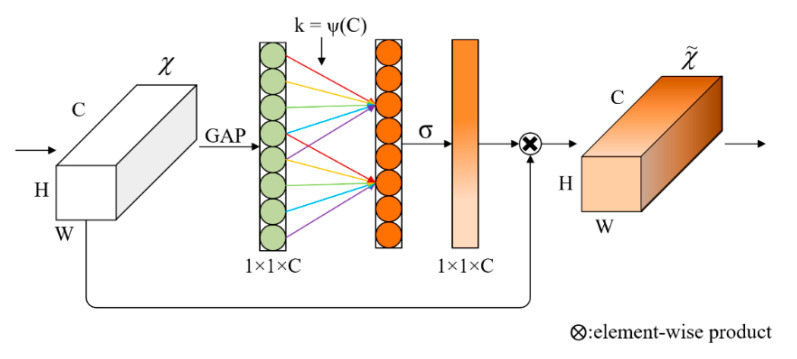
ECA-Net structure.

The structure of ECA-Net is shown in Figure 9, where the input is a feature tensor χ of H × W × C. A feature vector of size 1 × 1 × C is obtained after a GAP (global average pooling) operation, and then the weights are obtained by sliding convolution of a 1D convolution kernel of length *k* followed by a Sigmoid function output: again, the original input feature map is multiplied element by element with the weights to obtain the final output $\tilde{\chi }$. The ECA-Net is less computational and parametric than SE-Net and was added to the network model to improve accuracy, with little impact on the network’s computational speed.

Qin et al. [17] (the authors of FCA-Net) argued that global average pooling (GAP) is a special case of the 2D discrete cosine transform (DCT) based on frequency analysis:

(6) $$f_{h,\omega }^{2d}=\overset{H-1}{\underset{i=0}{\sum }}\overset{W-1}{\underset{j=0}{\sum }}x_{i,j}^{2d}B_{h,\omega }^{i,j}$$

s.t. h ∈ {0, 1, H−1}, ω∈ {0, 1, W−1}, where $f^{2d}$ denotes the 2D DCT spectrum, $x^{2d}$ denotes the input 2D tensor, and *H* and W represent the height and width of the input tensor, respectively. *B* is the DCT basis function and has the following equation:

(7) $$B_{h,\omega }^{i,j}=\cos\left(\frac{\pi h}{H}\left(i+\frac{1}{2}\right)\right)\cos\left(\frac{\pi \omega }{W}\left(j+\frac{1}{2}\right)\right)$$

Then, it follows that:

(8) $$f_{h,\omega }^{2d}=\overset{H-1}{\underset{i=0}{\sum }}\overset{W-1}{\underset{j=0}{\sum }}x_{i,j}^{2d}\cos\left(\frac{\pi h}{H}\left(i+\frac{1}{2}\right)\right)\cos\left(\frac{\pi \omega }{W}\left(j+\frac{1}{2}\right)\right)$$

When *h* and ω are both 0, we have:

(9) $$\begin{array}{c} f_{0,0}^{2d}=\overset{H-1}{\underset{i=0}{\sum }}\overset{W-1}{\underset{j=0}{\sum }}x_{i,j}^{2d}\cos\left(\frac{0}{H}\left(i+\frac{1}{2}\right)\right)\cos\left(\frac{0}{W}\left(j+\frac{1}{2}\right)\right)\\ =\overset{H-1}{\underset{i=0}{\sum }}\overset{W-1}{\underset{j=0}{\sum }}x_{i,j}^{2d}\\ =gap\left(x^{2d}\right)HW \end{array}$$

From Equation (9), we can prove that GAP is a special case of feature decomposition by the lowest frequency in the frequency domain, and it was experimentally demonstrated [17] that the same network with the addition of channel attention modules that learn information in different frequency bands has higher accuracy for a network that learns low-frequency information in the channel. Therefore, we believe that the GAP operation has some justification to retain relatively better feature information. In the channel attention module, we still retained the GAP operation in the original structure.

## 5. Training and Testing the Network

The network was designed to segment the laser stripe emitted by the line laser transmitter and the background containing the various disturbances generated during the welding process, which is essentially a binary classification problem, so this work used the binary cross-entropy (BCE) loss function. When using the BCE loss function, a Sigmoid function needs to be added to the output layer as an activation function to convert the linear input to a non-linear output.

Evaluation metrics are generally calculated based on a confusion matrix [18,19] (shown in Table 4). In this paper, two metrics, MIoU and PA, were used to evaluate the segmentation results of the network model.

All code in this paper is in the Python programming language, and the neural network is trained in the Pytorch framework, with the specific software environment and hardware configuration shown in Table 5. The optimizer chooses SGD and trains for 50 epochs with different momentums, batch sizes, and learning rates to obtain parameters that enable the network to achieve higher accuracy. As shown in Table 6, the fixed learning rate was 0.001 in experiments 1–9. When the momentum was 0.8 and the batch size was 1, the network achieved higher accuracy. In experiments 10 and 11, different learning rates were selected, but the accuracy was still higher when the learning rate was 0.001. Therefore, when training the deep learning network model next, the specific training parameters were as follows: the optimizer was SGD, the momentum size was 0.8, the learning rate was fixed at 0.001, the mini-batch size was set to 1, and the total number of epochs was 200.

Keskar et al. [20] studied the quality of the model when different batch sizes were chosen for SGD, and they found that the quality of the model degrades when using larger batches and that training in smaller batches may introduce enough noise to the training to obtain a better model. The dataset is large, so the size of the video memory needs to be considered in training. Additionally, considering that in a practical scene, the input to the network is frame-by-frame images, there is no guarantee that the performance of a model trained in large batches will be unaffected in such scenes. Taking these factors into account, the values chosen when setting the mini-batch size were all lower.

## 6. Results

SegNet [21] and DFA-Net [22] are also commonly used networks with good performance in the segmentation domain. We trained ERF-Net, SegNet, and DFA-Net under the same conditions, and the final results are shown in Table 7. Our network model has relatively few parameters and little computation and has the highest MIoU. FLOPs are generally considered to represent network inference speed, but in practice, factors such as memory access are also taken into account [8], so the measurement of network speed also depends on the actual operation on a specific platform.

**Figure 10:**
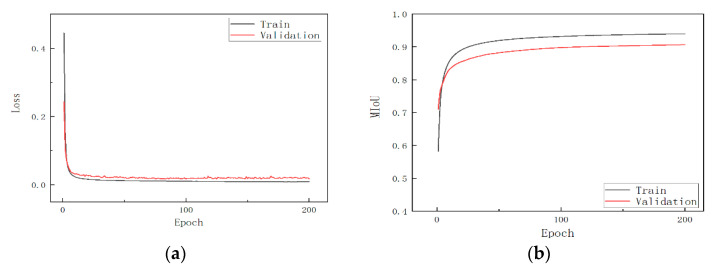
(**a**) Training loss and (**b**) MIoU change.

We measured the actual network segmentation speed on GPUs with different computing power resources, and it can be seen that our network model segments faster on the GTX 1050Ti, which has lower computational performance than other networks (SegNet is a special case). SegNet far exceeds the other networks in the table in terms of the inference speed, despite having more than ten times the FLOPs, in large part because the CUDNN library [23] has specialized optimizations for 3 × 3 convolutions, while the size of all of the convolutional kernels in the SegNet structure is 3 × 3. After training the network with the above parameters (the optimizer was SGD, the momentum size was 0.8, the learning rate was fixed at 0.001, the mini-batch size was set to 1, and the total number of epochs was 200), the loss curve and MIoU change curve of our network model are shown in Figure 10.

In this study, the Monte Carlo cross-validation (MCCV) method was used; i.e., the training set was mixed with the validation set and then randomly divided and trained in the same proportion to validate the segmentation performance of the improved network model. The training results are shown in Table 8. The improved network model in this paper did not appear to be overfitting after 200 epochs of training. The average MIoU was 0.9046, and the average PA was 0.9938.

## 7. Discussion

The network in this paper is designed for the application scene of weld feature stripe extraction and has only been tested on datasets collected at laser welding sites. We conclude that the network outperforms ERF-Net for weld seam feature extraction, mainly because (**a**) interference such as welding spatter around the weld feature stripes is a difficult part of the network for segmenting out the stripes, so a larger convolutional block is used in the encoding phase of the encoder to obtain more information about neighboring pixels for better learning by the deeper network; (**b**) the network is improved: it is lighter, uses more channels for more detailed classification in the third phase of encoding, and does not increase the size of the model. The addition of a channel attention mechanism allows the network to focus on channels containing laser feature stripes and to reduce the weight of channels containing splash interference and background; (**c**) improvements to the upsampling method allow for better restoration of the output image in the decoding stage, allowing for more accurate straight lines to be subsequently fitted based on the segmented stripes.

Although MIoU and PA are commonly used as metrics for evaluating segmentation networks, in the specific laser welding application scene of this paper, a larger proportion of the background portion of the segmentation resulted in much larger values for MIoU and PA in the final training of the network model applied to the scene in this paper than in the segmentation of other scenes. The increase in MIoU points, while directly reflecting an increase in segmentation accuracy, does not directly reflect the success of the segmentation of the network model in the application scene (weld feature stripe segmentation), i.e., whether the key weld feature points are segmented successfully. Failure of the weld feature segmentation will result in the weld point coordinates not being extracted, which in turn will affect the subsequent control of the weld gun trajectory by the welding robot. 

**Figure 11:**
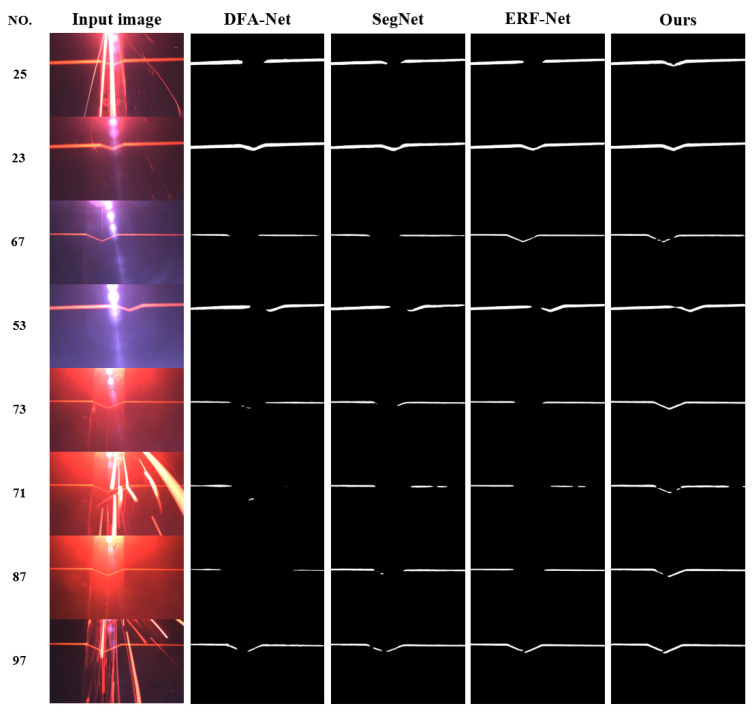
Comparison of the segmentation effects.

To this end, this study also used the simplest and most direct way to measure the performance of the network model; that is, after the network had segmented 100 images, the segmentation success rate of the welding feature points was compared. Due to the small number of images in the test set, we needed more images for testing to obtain a more accurate success rate, so we additionally re-collected another 100 images with varying degrees of interference at the welding site for testing. The segmentation effect was tested on 100 images, and a comparison of the segmentation effect of eight extracted images is shown in Figure 11.

**Figure 12:**
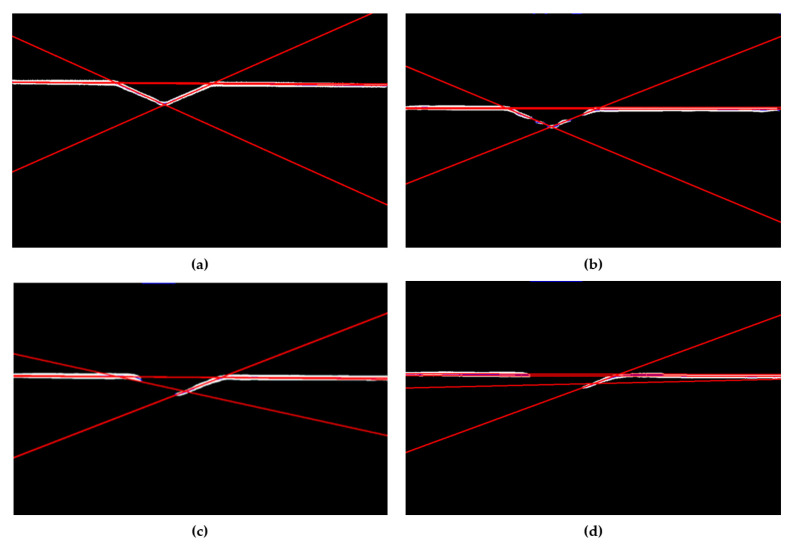
Extracted fringe centers and fitted straight lines: (**a**,**b**) segmentation succeeded and (**c**,**d**) segmentation failed.

From the figure, it can be seen that the network designed in this paper is more effective in segmenting the weld feature stripes compared to other similar networks. The segmentation can be considered successful when the center of the stripe can be extracted by the dynamic grey center-of-gravity method, the least-squares method can be used to fit a straight line (Figure 12), and the specific pixel coordinates of the weld feature points can be accurately calculated. In (**c**) of Figure 12, there is still a small streak below the intersection point, and it can be judged that the feature point is below the left side of the intersection point, and therefore, the segmentation fails. The final segmentation success rate results for each network are shown in Table 9.

After our network segmentation, it is almost always possible to complete a straight line fit and find the intersection point, although there are still some missing segments near the weld point. In contrast, the images segmented by other networks clearly show that most of the feature stripes near the critical weld points are missing in their entirety, making it impossible to continue to find the coordinates of the weld points. The network designed in this study achieved a success rate of 96% for the segmentation of 100 images, which is significantly higher than that of the other networks.

## 8. Conclusions

In this study, we designed a weld tracking module for real-time image acquisition by industrial robots during laser welding operations, and a total of 737 images were captured by this module. Of these, 637 images were first captured and labeled and then divided into a training set, a validation set, and a test set at a ratio of 8:1:1 for neural network training, and finally, 100 images were captured for the comparison of specific segmentation effects. The network that we designed has faster segmentation speed and higher segmentation accuracy than similar segmentation networks, with an average MIoU of 0.9046 after using the Monte Carlo cross-validation method. The inference speed is 82.1 FPS on a lower-performance GPU (GTX 1050Ti) for the segmentation of a 640 × 640 pixel size input tensor. In particular, in a practical comparison of the segmentation results, it is able to segment the weld feature stripes at important locations, and the segmentation success rate is much higher than that of ERF-Net, SegNet, and DFA-Net, with a 96% segmentation success rate.

## Figures and Tables

**Table 1:** Industrial camera-specific parameters.

Parameter Name	Parameter	Parameter Name	Parameter
Model	Sony IMX174	Image format	RGB
Sensor technology	CMOS	Frame rate	30FPS
Pixel size	5.86 μm	Operating temperature	0–50 °C
Resolution	1920 × 1200	Interface type	USB 3.0

**Table 2:** Dataset partitioning.

Category	Quantity	Proportion
Training set	509	80%
Validation set	64	10%
Test set	64	10%

**Table 3:** Network model structure.

	Layer	Type	Out-Channel	Out-Size
**ENCODER**	1	Downsampler block	16	320 × 320
	2	Downsampler block	64	160 × 160
	3–7	5 × Non-bt-1D-5	64	160 × 160
	8	Downsampler block	160	80 × 80
	9	DSConv-5	160	80 × 80
	10	Non-bt-1D-5 (groups 160)	160	80 × 80
	11–12	2 × Non-bt-1D-5 (dilated 2, groups 160)	160	80 × 80
	13	DSConv-5 (dilated 4)	160	80 × 80
	14	Non-bt-1D-5 (dilated 4, groups 160)	160	80 × 80
	15–16	2 × Non-bt-1D-5 (dilated 8, groups 160)	160	80 × 80
	17	DSConv-5 (dilated 16)	160	80 × 80
	18	Non-bt-1D-5 (dilated 16, groups 160)	160	80 × 80
	19–20	2 × Non-bt-1D-5 (dilated 32, groups 160)	160	80 × 80
	21	ECA-Net	160	80 × 80
	22	DSConv-5	160	80 × 80
	23	ECA-Net	160	80 × 80
**DECODER**	24	Interpolate block (upsampling)	64	160 × 160
	25–26	2 × Non-bt-1D-3	64	160 × 160
	27	Interpolate block (upsampling)	16	320 × 320
	28–29	2 × Non-bt-1D-3	16	320 × 320
	30	Deconvolution (upsampling)	2	640 × 640

**Table 4:** Confusion matrix.

Predicted Value	True Value	
	Laser Stripe	Background
**Laser stripe**	True positive (TP)	False positive (FP)
**Background**	False negative (FN)	True negative (TN)

**Table 5:** Software environment and hardware configuration.

Name	Parameter
System	Ubuntu 20.04.3 LTS (GNU/Linux 5.4.0-90-generic x86_64)
CPU	7-core Intel(R) Xeon(R) CPU E5-2680 v4 @2.40 GHz
GPU	NVIDIA GeForce RTX 3090 ∗ 1, VRAM: 24 GB
Environment configuration	PyTorch 1.10.0 + Cuda 11.3 + Python 3.8

**Table 6:** Accuracy achieved with different parameters.

No.	Momentum	Lr	Batch Size	MIoU	PA
**1**	0.7	0.001	1	0.860561	0.989663
**2**	0.7	0.001	4	0.665455	0.965270
**3**	0.7	0.001	8	0.608225	0.956638
**4**	0.8	0.001	1	0.881789	0.992213
**5**	0.8	0.001	4	0.749245	0.981168
**6**	0.8	0.001	8	0.721268	0.943954
**7**	0.9	0.001	1	0.852441	0.990590
**8**	0.9	0.001	4	0.864416	0.990858
**9**	0.9	0.001	8	0.773669	0.978236
**10**	0.8	0.005	1	0.844770	0.990292
**11**	0.8	0.0002	1	0.711708	0.972816

**Table 7:** Network performance comparison.

Model	MIoU	PA	Parameters	FLOPs	GTX 1050Ti	RTX 3090
**ERF-Net**	0.898	0.993	2.06 M	23.03 G	74.9 FPS	78.7 FPS
**SegNet**	0.887	0.992	29.44 M	250.87 G	143.7 FPS	154.8 FPS
**DFA-Net**	0.842	0.990	2.36 M	3.39 G	23.4 FPS	26.0 FPS
**Ours**	0.906	0.993	0.75 M	15.41 G	82.1 FPS	87.9 FPS

**Table 8:** Monte Carlo cross-validation.

No.	Validation Set MIoU	Validation Set PA	Test Set MIoU	Test Set PA
**1**	0.906	0.9936	0.907	0.9939
**2**	0.898	0.9940	0.889	0.9926
**3**	0.906	0.9945	0.884	0.9924
**4**	0.914	0.9945	0.895	0.9933
**5**	0.899	0.9926	0.885	0.9928

**Table 9:** Network segmentation success rate.

Model	Segmentation Success Rate (%)
**ERF-Net**	82
**SegNet**	66
**DFA-Net**	77
**Ours**	96

## Data Availability

The data are not publicly available due to the company requirements.
